# Breast cancer in European Union: An update of screening programmes as of March 2014 (Review)

**DOI:** 10.3892/ijo.2014.2632

**Published:** 2014-09-01

**Authors:** E. ALTOBELLI, A. LATTANZI

**Affiliations:** 1 Department of Life, Health and Environmental Sciences, University of L’Aquila, L’Aquila, Italy; 2 Epidemiologic and Social Marketing Unit, AUSL 4 Teramo, Italy

**Keywords:** cancer, breast, screening, EU28

## Abstract

Breast cancer, a major cause of female morbidity and mortality, is a global health problem; 2008 data show an incidence of ~450,000 new cases and 140,000 deaths (mean incidence rate 70.7 and mortality rate 16.7, world age-standardized rate per 100,000 women) in European Union Member States. Incidence rates in Western Europe are among the highest in the world. We review the situation of BC screening programmes in European Union. Up to date information on active BC screening programmes was obtained by reviewing the literature and searching national health ministries and cancer service websites. Although BC screening programmes are in place in nearly all European Union countries there are still considerable differences in target population coverage and age and in the techniques deployed. Screening is a mainstay of early BC detection whose main weakness is the rate of participation of the target population. National policies and healthcare planning should aim at maximizing participation in controlled organized screening programmes by identifying and lowering any barriers to adhesion, also with a view to reducing healthcare costs.

## 1. Introduction

Breast cancer (BC) is a global health problem and one of the principal causes of female morbidity and mortality (1–3). Its distribution (incidence, prevalence) and the economic burden it imposes on national health services make it a major public health concern both in developed and developing countries (4).

BC is the most common neoplasm affecting women aged <45 years and is even more prevalent in the 45–65-year age group. BC is the main cause of female death from cancer worldwide.

In 2008 ~1.4 million new BC diagnoses were made throughout the world and 446,000 women died of BC; in the same year incidence in EU Member States (EU28) was ~450,000 new cases with 140,000 deaths, accounting for a mean incidence rate of 70.7 and a mean mortality rate of 16.7 per 100,000 women (world age-standardized rate, ASR-W) (5).

**Figure 1:**
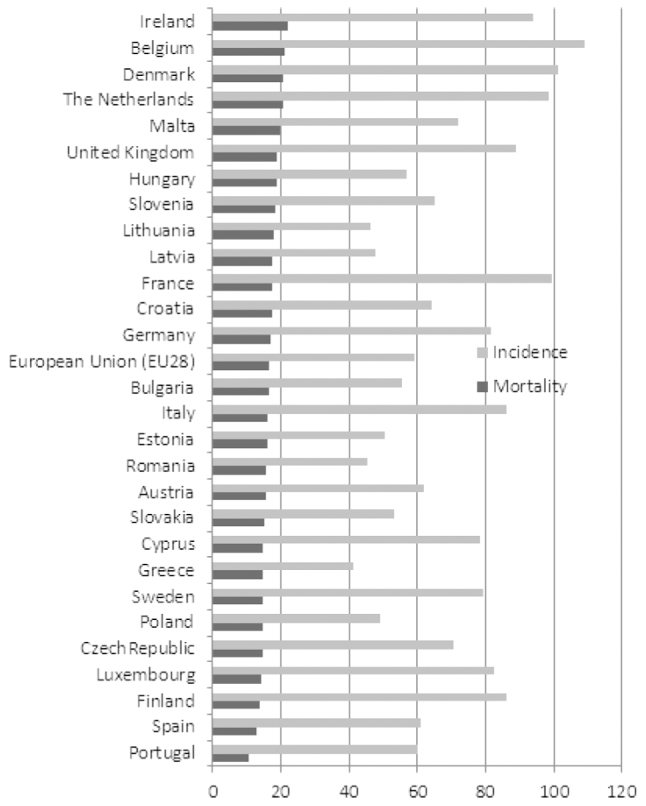
Breast cancer incidence and mortality in the European Union (EU28). ASR-W, world age-standardized rates per 100,000.

Different incidence, mortality and survival rates are due to different risk factors, availability of organized screening programmes, and access to effective treatment (1). Despite the fact that mortality tends to be higher in less prosperous countries (Fig. 1) (6), the incidence of BC in Western Europe is among the highest in the world (5) and BC prevention is a major public health goal also in the EU.

Retrospective studies of death causes among females over the past 30 years based on World Health Organization data in the majority of European countries have reported a variety of situations with considerable changes in BC mortality rates, including a ~40% reduction in Ireland and a 17% increase in Romania. The most developed North-Western European countries all experienced reductions, while increments were recorded in Central European States (Table I). The highest reductions involved women aged <50 years also in areas lacking active screening programmes; reductions were less striking among 50- to 69-year olds, and a greater variability, even including strong increments, was found among women older than 70 years (7–10).

An increased BC incidence and a peak in the 1980s in all countries was followed by a reduction in BC mortality both in countries adopting screening programmes and in those lacking them, probably due to advances in surgical techniques and treatments in all countries (11,12) (Table I).

The favourable effects of organized screening are however well established. A number of trials have shown large reductions in mortality (13–17) that in a study carried out in the Netherlands actually reached 70% (18); despite some possible biases in showing the mortality reduction, all have consistently demonstrated the effectiveness of screening in reducing BC mortality (19).

In 2003 the European Council recommended the implementation of cancer screening programmes based on European best-practice guidelines (20).

According to the International Agency for Cancer Research, participants in organized screening programmes, where 50–69-year-old women are invited to undergo mammography at 2-year intervals, are 35% less likely to die from BC (21). According to a more recent review of methodologically more stringent studies the probable impact of EU28 screening programmes on women invited to screen is a 26% reduction in mortality (95% confidence interval, CI, 13–36 %) at 6–11-year follow-up (22).

Despite widespread agreement among EU Member States on the important role of population-based (PB) screening in controlling cancer, the 2007 EU report reviewing compliance with Council recommendations found that several states still had no BC screening programme. We review the progress of EU28 screening programmes as of March 2014.

## 2. Screening techniques

Clinical breast examination (CBE) is the basic physical examination of the breast, especially in symptomatic women. In the French programme it is an integral part of first-level screening (23), guiding in its performance and interpretation. Albeit carried out by experienced professionals integration with other tests is required in presence of suspicious finding or sign.

Mammography is the sole screening method recognized by the European Commission for women aged 50–69 years. It is the morphological method enabling examination of the breast in its entirety and offering the highest sensitivity also for early-stage tumours, especially in women with predominantly dense breasts. Its specificity is affected by breast density and reporting technique.

A review of the European literature examining the impact of mammographic screening on BC mortality found a reduction of 25–31% among invited women and of 38–48% among those who actually screened (24). According to the authors the reason for the debate on BC screening is the adoption of analytical methods unsuitable to capture the real effects of screening.

An independent British study confirmed these data and found an ~20% reduction in BC mortality among women subjected to screening, with ~1% overdiagnosis (25). However further analysis of the data led the authors to state that overdiagnosis rates cannot be quantified with precision. Views on how to evaluate the risk of overdiagnosis differ, resulting in estimates that can range from 0 to 50% depending on the method used (26).

Overdiagnosis has been attracting growing interest; the issue is closely related to the availability of sensitive diagnostic tests and to the high probability of detecting slow-growing or non-aggressive lesions. It is a useful parameter to assess the impact of screening on overall female health and is evaluated by comparing tumour incidence in women screened for a given period and in women who have never been screened.

An Italian study based on cancer registry data assessed the effect of BC screening on the reduction of diagnoses of highly invasive lesions. Organized programmes that had been active for several years were associated with a significant, stable reduction in the incidence of pT4 lesions and with an increase in pT2 lesions starting in the 3rd-4th year of the programme, with an incidence rate ratio that decreased from 0.81 (95% CI, 0.75–0.88) to 0.71 (95% CI, 0.64–0.79) in the 7th-8th year (27).

Finally, a large number of diagnostic centres have adopted digital mammography, which has improved diagnosis and enables better scan management and storage. The image is shown in real time on high-resolution monitors and is later archived in electronic image filing systems. The adoption of digital mammography in 2007 rapidly resulted in a doubling of referral rates in the Netherlands screening programme (28); moreover, the improved equipment sensitivity resulted in a reduction in false-positives. Computer-assisted techniques combined with digital mammography also enhance lesion detection (29).

## 3. Breast cancer screening: spontaneous or organized?

In 2003 the European Commission recommended PB screening for women aged 50–69 years; in 2007 programmes based on Council indications were active or were being organized in 22 states.

Alternatives to organized screening, proposed with a view to achieving earlier diagnosis, encourage self-referral to breast units interconnected on line, also with a view to monitoring the quality of examinations. These models exploit and improve existing diagnostic resources and facilities and aim at tailoring diagnostic and clinical protocols to the risk profile and clinical condition of each subject; moreover they are useful in settings where it is difficult to use classic screening methods with individual invitations. However, several national and European experiences indicate that screening by invitation achieves high levels of coverage more rapidly and that the cost of organized programmes is more limited (30–33).

## 4. The situation in EU28

**Figure 2:**
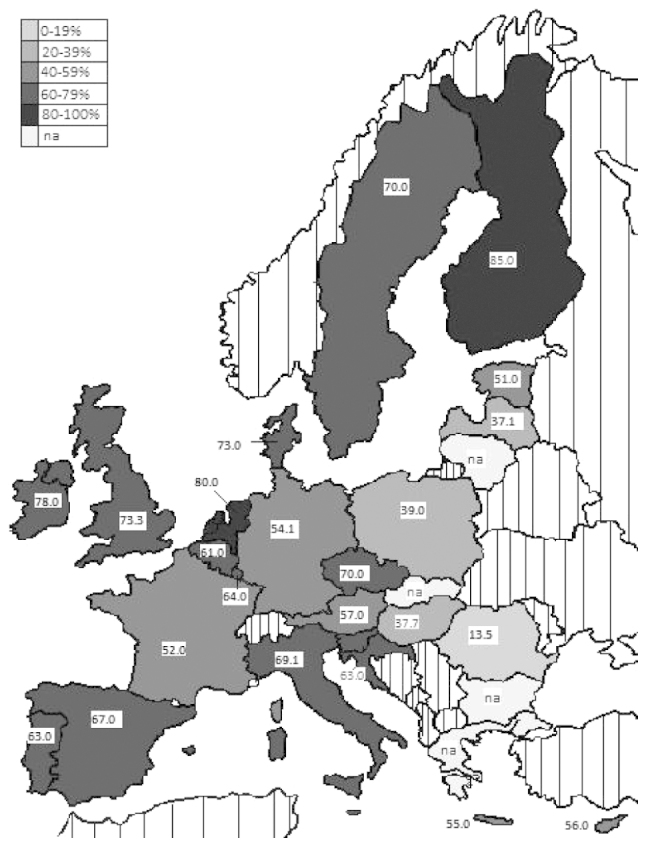
Distribution of breast cancer screening programmes in EU28 and coverage rates. Participation data are divided into five classes from 0 to 100%. Participation rates are those reported in Table II. Member states for which attendance data were not available are indicated with NA.

The current situation of screening programmes in EU28 is described below and reported in Table II and shown in Fig. 2. The present study is based on the most recent data available from PubMed-indexed journals, the websites of the Health ministries of each member state, and the websites of national cancer observatories; failing these sources, information was sought in scientific journals published in the local language.

In 1974 Austria was the first EU Member State to implement a BC screening programme. Screening remains opportunistic in most of the country, with mammography offered to all women >40 years old (34). In Tyrol an organized PB programme offering a yearly mammogram to women with healthcare insurance aged 40–49 years and biannual examination to those aged 60–69 years has been active since 2007; it does not envisage double reading of mammograms (35).

With 147.5 cases per 100,000 women, Belgium has the highest BC incidence in the world (36). Its organized screening programme offers mammograms every 2 years to women aged 50–69 years. However, opportunistic screening is quite widespread, and ~80% of diagnostic mammographic examinations are believed to be related to spontaneous screening. Moreover 85% of examinations are combined with ultrasound (US) scanning performed on the same day; this may indicate that especially in Wallonia and in Brussels US is used as a screening method. This in turn may account for the large number of second-line examinations (biopsy and fine needle aspiration) performed each year (37). Attendance is ~61% according to a 2010 survey (38).

Bulgaria has no active national programme. BC prevention is entrusted to a private association, the Centre for Protection of Rights in Health Care, which since 2011 has been conducting screening examinations with mobile units throughout the country. The local authorities in the municipalities visited are in charge of making appointments for women aged 40–60years who wish to be examined (39).

In 2006 Croatia set up an organized screening programme offering biannual mammograms at various sites (public hospitals, universities, private facilities) (40,41). A recent quality audit of sample mammograms highlighted severe problems in breast positioning and lesion detection that had the potential to affect screening effectiveness and that could be addressed by improving personnel training and operative strategies (42).

Cyprus set up its first pilot programme in Nicosia in 2003; the programme was extended nationwide in 2006 (43). It is a centralized PB screening programme that is offered to women aged 50–69 years. The Health Ministry website contains information on the shift to digital mammography (44).

The Czech Republic does not have a centralized PB screening programme. Physicians and gynaecologists advise women aged 45–69 to undergo a free examination; in 2010 the upper age limit was removed. Mammograms are taken with traditional or digital machines (45). The results are archived in a national database that is accessible online (www.svod.cz) and allows monitoring BC trends at both the national and regional level, also providing several reference parameters. Screening adhesion is high (46).

For many years screening programmes have been in place in few areas of Denmark, Copenhagen (1991) and Fyn (1993) being the first areas to be served. Nationwide coverage was achieved in 2010. Mammograms are offered to women aged 50–69 years (47). For a long time the first round offered two views and the second a single view; the two views have subsequently been extended to the second round. The reduction in mortality found in areas offering screening (12) has raised controversy because similar rates seem to be found in areas not offering it (47,48).

Estonia made a large effort since it implemented a BC screening programme in 2002. At first the target population was limited to 45–59 year olds with healthcare coverage, but since 2007 mammograms are offered to all 50–65-year-olds. Digital mammography was introduced in 2006 (49). The +9.6% mortality rate (12) needs careful assessment by comparison with a study reporting a rate of +25.5% in 1990–2002 (50).

In Finland BC screening is managed by local authorities, who are responsible for the activation, delivery and quality assessment of services, which can be provided autonomously or be outsourced from public or private bodies. The central government evaluates service quality through a team of experts. According to the Health and Social Services Ministry website (51) participation in 2009 was 84% compared with the OECD mean of 62%.

In France women aged 50–74 are offered mammograms every 2 years followed by CBE. Screening is by invitation; digital mammography was introduced in 2008. France is the first EU country by volume of yearly screening mammograms (52).

In Germany a number of pilot projects were followed in 2005 by activation of the national programme. The national centre invites women aged 50–69 years to screen every 2 years (53).

Greece has the lowest BC incidence in EU28 but a rising mortality rate. Screening is exclusively opportunistic and attendance is unknown (54). Some sporadic pilot projects have been active since the 1990s.

In Hungary the organized programme, implemented in 2002, is paralleled by strong spontaneous screening. Although adhesion to the organized programme is on the rise (53.5% in 2005), >350,000 women use non-organized screening (55).

Ireland introduced screening in 2000 and slowly extended it nationwide. The target population is the 50–69-year age group; the examination is offered every 2 years. Digital mammography was adopted nationwide in 2008. The use of mobile units is widespread (56).

In Italy PB screening began in 1990; nationwide coverage was attained in 2007. The target population is generally aged 50–69 years, but in some regions it includes 45-year-olds and in others women aged ≤74 years are also invited. The mean rate of adhesion to the various programmes is 60.5%. Spontaneous screening is not easy to quantify, but a 2010 survey (project PASSI) found that 61% of women aged 40–49 years had undergone at least one preventive mammogram (57).

Latvia activated screening for BC and uterine cancer in 2009. The management of invitations is centralized and is based on the population registry. The equipment is generally analogical but some facilities have digital machines. Participation, poor at first, is slowly rising: at the end of October 2013 it was 37%. The Health Ministry website (58) provides updated information on the activity of mobile units and attendance rates.

Lithuania activated a screening programme in 2005 but could not implement it nationwide due to lack of facilities and specialized personnel. No information is available on its progress (59). The mortality rate does not seem to have changed over the last 20 years.

Luxembourg adopted a programme with centralized management of invitations and reminders in 1992 (60). In 2001 it began to adopt digital equipment to archive images and enable double reading also remotely (61).

In Malta screening was implemented in 2009. The Health Ministry provides all the necessary information on the project but it may have planned poorly, because Malta boosts one of the worst records in terms of the number of mammograms taken within national screening programmes (5%); in contrast private centres, which 50% of the population have visited at least once, are quite busy (46).

In the Netherlands screening began in 1989. Women aged 50–69 years are screened at 2-year intervals and those aged ≤74 years were added in 1998. Two views are taken in the first round and a single view thereafter. Nationwide adoption of digital mammography was completed in 2010 (28).

In 2007 Poland set up a centralized PB programme offering digital mammograms to women aged 50–69 years at 2-year intervals, except those undergoing follow-up (62). Radiologists at all levels use computer-assisted techniques to improve diagnostic performances (63).

Portugal implemented its first region-based screening programme in 1990; nationwide screening was achieved in 2005. The Health Ministry aims at 60% coverage by 2016 (64). The programme offers digital mammography to women aged 45–69 years, also with mobile units.

In Romania, which has no screening programme, not for profit organizations help the government increase cancer awareness and prevention and provide healthcare and screening on request. The Romanian Cancer Society (65) recommends screening mammograms at 3-year intervals from age 40 years, biannual examination between 45 and 50 and yearly screening thereafter.

In 2008 Slovakia began to organize a BC screening programme that has not yet been activated. Even though prevention is merely opportunistic, 80% of women have been examined at least once in their life (46).

In Slovenia the PB DORA screening programme, introduced in 2008, has been geared to achieve nationwide coverage over a few years. Women aged 50–69 years are invited to screen at mobile units or at the Ljubljana cancer centre. The response has been very good, exceeding a participation rate of 75% after the third screening round (66).

Spain introduced a BC screening programme in Navarre in 1990 and achieve national coverage in 2009. Digital mammography is spreading. The target population (aged 50–69 or 45–69 years in different programmes) is invited to screen at 2-year intervals (67).

Sweden has one of the first programmes introduced in Europe, but exhibits considerable organizational variability. Biannual mammograms are generally offered to women aged 50–69 years, but in >60% of the country women aged 40–49 years are also invited to test every 18 months; in about half of the country 70–74-year-olds are also offered a biannual mammogram.

The UK has different PB programmes in England, Wales, Scotland and Northern Ireland, with testing usually offered at 3-year intervals. In Northern Ireland the screening programme, introduced in 1990, was initially aimed at 50–64-year-olds but was extended to 70-year-olds in 2004. In Scotland the target population is aged 50–70 years. The shift to digital mammography is ongoing. Wales activated its programme in 1989 (50–70-year-olds). It adopted digital mammography in 2011. In England the shift is nearly complete (68). Here a trial begun in 2010 is assessing the value of extending BC screening to 47–73-years-olds (69). Mortality in 1989–2006 fell by 35% in England and Wales, by 30% in Scotland and by 29% in Northern Ireland (12).

## 5. Discussion

BC is probably a heterogeneous group of diseases with distinct natural histories. The notion that cancer progresses inexorably from atypia to carcinoma *in situ*, invasive carcinoma and then metastasis is no longer tenable (70–74).

Early diagnosis of abnormalities is increasingly important to gain a greater understanding of the risk of progression of the individual lesions and of the disease in general. Crucial issues for screening programmes involve the management of such abnormalities to improve survival and the interval between examinations (21). We review the epidemiological scenario of BC, active screening programmes, and changes in EU28 mortality rates.

The introduction of a cancer screening programme entails an increased rate of diagnoses; any changes to a screening programme that has been active for some years may also have an impact on incidence. For instance, the introduction of digital mammography in the Netherlands led to a strong increase in BC diagnosis but has not so far affected mortality. The mortality data tend to be more stable over time, and their change is related to a variety of disease-related factors. Examination of BC mortality rate changes shows that reductions also occurred in countries that set up a screening programme after 2006. This is explained by the survival-enhancing effect of more effective diagnostic methods, surgical techniques and treatments (11).

Overall, screening programmes offer the advantage of early lesion detection, enabling their management before progression and worsening.

Adhesion to BC screening, like participation in colorectal tumour screening (75), is still among the weaknesses of the programmes adopted by several EU28 countries.

The target rate of participation of 75% is not achieved several states. Poor knowledge of the disease and of the attendant risk (76) as well as organizational barriers (e.g., test hours coinciding with work hours; the need for reaching facilities far away from one’s residence) may significantly limit participation, especially in less prosperous countries. This situation does not meet one of the main criteria of screening programmes, their ethics, since all women should have equal access to cancer screening and to quality treatment and post-treatment care irrespective of place of residence, social standing, job and education. Yet marked disparities are currently found among member states, regions and even hospitals in the same area.

In this light the 2008 Commission Report focuses on the implementation of Council recommendations by aiming at reducing disparities among states by promoting the sharing of the best experiences and abilities gained. To do this, capillary diffusion of appropriate information systems capable of evaluating cancer trends in the population, like cancer registries, is required. Such tools are highly cost-effective, since important information on cancer diagnosis/treatment is provided at small cost to the healthcare service, enabling identification and management of any weaknesses, especially in problems areas and programmes. Full exchange and circulation of information among member states would enable the system to be completed and EU collaboration to flourish.

## 6. Conclusions

Even though the European scenario currently requires a curb on public spending, the various national health services should guarantee screening access and participation to the largest possible number of subjects, also considering that adhesion is a weakness of many programmes. Awareness campaigns and training of healthcare providers may be a good and economical starting point to improve the knowledge of disease risk and enhance screening compliance also in the short term; at the same time the cancer registries system would allow monitoring the effectiveness of the fight against cancer also in the light of the fact that population ageing entails a constant increase in the incidence of these diseases.

## Figures and Tables

**Table I:** Breast cancer standardized mortality and incidence rates in European Union Member States and changes in mortality rates from 1989 to 2006.

Member state	Mortality rate^a^	Incidence rate^a^	Change in mortality %
Austria	15.4	62.1	−26.8 (7)
Belgium	21.0	109.2	−24.6 (12)
Bulgaria	16.5	55.5	−0.8 (7)
Croatia	17.6	64.0	−0.3 (41)
Cyprus	14.9	78.4	NA
Czech Republic	14.5	70.8	NA
Denmark	20.8	101.1	−17.8 (7)
Estonia	15.9	50.2	−20.8 (7)
European Union (EU28)	16.7	63.1	+9.6 (7)
Finland	13.7	86.1	−11.7 (7)
France	17.6	99.7	−10.7 (7)
Germany	16.9	81.8	−21.3 (53)
Greece	14.9	41.4	+1.4 (7)
Hungary	18.6	56.8	−11.4 (7)
Ireland	21.8	93.9	−26.7 (12)
Italy	16.1	86.3	−22.8 (7)
Latvia	17.6	47.9	+11.4 (7)
Lithuania	17.8	46.4	−0.7 (7)
Luxembourg	14.2	82.3	−34.1 (7)
Malta	19.6	72.2	NA
Poland	14.7	48.9	−25.0 (12)
Portugal	10.7	60.0	−5.9 (7)
Romania	15.6	45.4	+17.8 (7)
Slovakia	15.1	53.4	+16.6 (7)
Slovenia	18.4	64.9	−1.5 (7)
Spain	12.9	61.0	−16.1 (7)
Sweden	14.8	79.4	−26.8 (7)
The Netherlands	20.5	98.5	−16.8 (12)
United Kingdom	18.6	89.1	−29.6/−35 (46)

Mortality and incidence rate are adapted from ref. 5.

a Per 100,000 inhabitants.

NA, data not available.

**Table II:** Distribution of cancer screening programmes in EU28 as of March 2014.

Region/member state	Programme type and extension		Screening method in use	Views	Double	Screening interval reading (years)	Age of target population	Programme start date	Natw coverage	Attendance in 2010 (%) (46)
Austria	NPB	Natw	Fm	2	No	2	>40	1974	-	NA
	PB	Reg^a^	Dm	2		1/2	40–59/60–69	2007	2008	57.0 (35)
Belgium	PB	Natw	Dm	2	Yes	2	50–69	2001	-	61.0 (12)
Bulgaria	NPB	Local	Fm	-	-	-	45–69	2011	-	NA
Croatia	PB	Natw	Fm Dm	2	Yes	2	50–69	-	2006	63.0
Cyprus	PB	Natw	Dm	2	Yes	2	50–69	2003	2006	56.0 (43)
Czech Republic	NPB	Natw	Fm Dm	2	Yes	2	45–69	2002	2007	70.0 (46)
Denmark	PB	Natw	Dm	2	Yes	2	50–69	1991	2010	73.0
Estonia	PB	Natw	Dm	-	-	2	50–65	2002	2007	51.0
Finland	PB	Natw	Dm	2	Yes	2	50–69	1987	1989	85.0
France	PB	Natw	Fm Dm CBE	2	Yes	2	50/74	1989	2004	52.0
Germany	PB	Natw	Fm Dm	2	Yes	2	50–69	2005	2009	54.1
Greece	NPB	Pilot	Fm	2	-	1/2	40–50/64	-	-	NA
Hungary	PB	Natw	Fm	2	Yes	2	45–65	2002	-	53.5 (55)
Ireland	PB	Natw	Dm	2	Yes	2	50–64	2000	2008	78.0 (12)
Italy	PB	Natw	Fm Dm	2	Yes	2	50–69 (74)	1990	2007	69.1
Latvia	PB	Natw	Fm Dm	2	No	2	50–69	2008	2009	37.1
Lithuania	PB	Natw	Fm	2	Yes	2	50–69	2005	-	NA
Luxembourg	PB	Natw	Dm	2	Yes	2	50–69	1992	1992	64.0
Malta	PB	Natw	Dm	2	-	3	50–59	2008	2009	55.0 (46)
The Netherlands	PB	Natw	Dm	2 (1)	Yes	2	50–74	1988	1997	80.0
Poland	PB	Natw	Dm	2	Yes	2	50–69	2006	2007	39.0
Portugal	PB	Natw	Dm	2	Yes	2	45–69	1990	2005	63.0
Romania	NPB	Local	Fm	2	No	(3) (2) 1	40+	-	-	13.5 (46)
Slovakia	NPB	-	-	-	-	2	40+	-	-	NA
Slovenia	PB	Natw	Dm	2	Yes	2	50–69	2008	-	75
Spain	PB	Natw	Fm Dm	2	Yes	2	(45) 50–69	1990	2009	67.0
Sweden	PB	Natw	Fm Dm	2	Yes	(1.5) 2	40 (50)–(69) 74	1986	1996	70.0
United Kingdom	PB	Natw	Fm Dm	2	No	3	50–(64) 70	1988	1995	73.3

PB, population-based; NPB, non-population-based; Natw, nationwide; Reg, regional; Local, limited to some municipalities; Fm, screen-film mammography; Dm, digital mammography; CBE, clinical breast examination; NA, data not available.

a Target population includes women living in Tyrol region.
